# Ritonavir-boosted protease inhibitor monotherapy is 6% less effective than combination antiretroviral therapy in a meta-analysis

**DOI:** 10.1186/1758-2652-13-S4-O18

**Published:** 2010-11-08

**Authors:** WFW Bierman, EH Humphreys, MA van Agtmael, CA Boucher, GW Rutherford

**Affiliations:** 1 University Medical Centre Groningen, Internal Medicine, Groningen, Netherlands; 2 University of California, San Francisco, Global Health Sciences, San Francisco, USA; 3 VU University Medical Centre, Internal Medicine, Amsterdam, Netherlands; 4 University Medical Centre Rotterdam, Virology, Rotterdam, Netherlands

## Background

Ritonavir-boosted protease inhibitor (PI/r) monotherapy has several potential benefits over standard PI-based combination antiretroviral therapy (cART) and has recently been included in the European HIV treatment guidelines. However, there are concerns about its efficacy. We performed a meta-analysis comparing PI/r monotherapy to cART to examine its efficacy.

## Methods

We searched electronic databases (Pubmed, EMBASE, Central) from 1996 to 2010 using keywords, "protease inhibitor", "antiretroviral", "monotherapy", relevant drug names and standard "HIV" and "RCT" search strings on March 22, 2010, without limits to language. We searched major HIV-related conferences manually from 2007 and contacted experts. Two reviewers independently assessed citations for eligibility and extracted relevant data. Assessment of bias of individual studies was performed independently by both reviewers. We did not include review articles, single-arm trials, or observational studies.

## Results

Of the 137 citations identified, we reviewed 19 articles after duplicates and obviously unrelated titles were discarded. Of these, 6 met eligibility criteria. Four additional abstracts were identified from conference abstracts. Ten RCTs (1265 participants) were included in meta-analysis using random effects Mantel-Haenszel methods. Summary estimate for the outcome of viral suppression (<50 copies/ml) on PI/r monotherapy compared to cART using intention to treat analysis (ITT), where reinduction and missing data count as failure, was RR_MHRE_ 0.94 (95% CI 0.89-0.99) without evidence of heterogeneity (p-value 0.55 and I^2^ 0%). Using on-treatment (OT) analysis, where missing information, deaths and drug changes due to adverse events were censored, the summary estimate was RR_MHRE_ 0.90 (95% CI 0.85-0.96, 1113 participants). There was no evidence of statistical heterogeneity in the OT analysis (p-value 0.01, I^2^ 57%). All studies were open-label. There was variability in study populations (treatment naïve vs experienced) and in interventions (LPV/r vs DRV/r monotherapy and cART regimens). Excluding the only RCT that started PI/r monotherapy in patients with unsuppressed HIV, did not change the ITT summary estimate.

## Conclusions

Our meta-analysis of 10 RCTs suggests that PI/r monotherapy is slightly but significantly less effective than cART. Subgroups of patients, however, may benefit from this promising alternative treatment strategy. Future RCTs should focus on these patients.

